# Pediatric kidney transplants with multiple renal arteries show no increased risk of complications compared to single renal artery grafts

**DOI:** 10.3389/fped.2022.1058823

**Published:** 2022-12-16

**Authors:** Juliano Riella, Marina M. Tabbara, Angel Alvarez, Marissa J. DeFreitas, Jayanthi Chandar, Jeffrey J. Gaynor, Javier González, Gaetano Ciancio

**Affiliations:** ^1^ Department of Surgery, University of Miami Miller School of Medicine, Jackson Memorial Hospital, Miami, FL, United States; ^2^ Miami Transplant Institute, University of Miami Miller School of Medicine, Jackson Memorial Hospital, Miami, FL, United States; ^3^ Department of Pediatrics, Division of Nephrology, University of Miami Miller School of Medicine, Jackson Memorial Hospital, Miami, FL, United States; ^4^ Servicio de Urología, Hospital General Universitario Gregorio Marañón, Madrid, Spain; ^5^ Department of Urology, University of Miami Miller School of Medicine, Jackson Memorial Hospital, Miami, FL, United States

**Keywords:** pediatric kidney transplantation, multiple renal arteries, surgical technique, vascular reconstruction, liver mobilization technique

## Abstract

**Background:**

Kidney allografts with multiple renal arteries (MRA) are not infrequent and have been historically associated with a higher risk of developing vascular and urologic complications. Reports of kidney transplantation using MRA allografts in the pediatric population remain scarce. The aim of this study was to evaluate if transplantation of allografts with MRA with a surgical intent of creating a single arterial inflow using vascular reconstruction techniques when required, and without the routine use of surgical drains or ureteral stents, is associated with an increased risk of complications when compared to single renal artery (SRA) grafts.

**Methods:**

We retrospectively analyzed all pediatric renal transplant recipients performed by a single surgeon at our center between January 2015 and June 2022. Donor and recipient demographics, intraoperative data, and recipient outcomes were included. Recipients were divided into two groups based on SRA vs. MRA. Baseline variables were described using frequency distributions for categorical variables and means and standard errors for continuous variables. Comparisons of those distributions between the two groups were performed using standard chi-squared and *t*-tests. Time-to-event distributions were compared using the log-rank test.

**Results:**

Forty-nine pediatric transplant recipients were analyzed. Of these, 9 had donors with MRA (Group 1) and 40 had donors with SRA (Group 2). Native kidney and liver mobilization was performed in 44.4% (4/9) of Group 1 vs. 60.0% (24/40) of Group 2 cases (*p* = 0.39). There were no cases of delayed graft function or graft primary nonfunction. No surgical drainage or ureteral stents were used in any of the cases. One patient in Group 2 developed a distal ureter stricture. The geometric mean serum creatinine at 6- and 12-months posttransplant was 0.7 */ 1.2 and 0.9 */ 1.2 mg/dl in Group 1 and 0.7 */ 1.1 and 0.7 */ 1.1 mg/dl in Group 2. Two death-censored graft failures were observed in Group 2, with no significant difference observed between the two groups (*p* = 0.48).

**Conclusions:**

Our study demonstrates that pediatric renal transplantation with MRA grafts, using a surgical approach to achieve a single renal artery ostium, can be safely performed while achieving similar outcomes as SRA grafts and with a low complication rate.

## Introduction

Renal transplantation remains the best therapy for the pediatric population that suffers from end-stage kidney disease (ESKD), offering improved development and growth (1). Based on a report released by the Scientific Registry of Transplant Recipients (SRTR), there were 2,637 pediatric candidates on the waiting list, with more than 1,000 new candidates added to the list in 2020 (2). There is still a high discrepancy between demand and organ availability; however, advancements in surgical techniques have helped transplant surgeons perform complex vascular reconstructions and utilize grafts with multiple renal arteries (MRA) without an increased risk of vascular or urological complications (3, 4).

When evaluating pediatric kidney transplant (KT) results, data remain scarce, and few publications have demonstrated the safety of using renal grafts with MRA (3). In their recent retrospective study of 379 pediatric transplants, 24% of which contained MRA, O’Kelly et al. (3) demonstrated no significant differences in the incidence of *postoperative complications* up to 3 months, estimated glomerular filtration rate (eGFR) and renal function up to 1 year, and graft survival up to 4 years posttransplant, irrespective of allograft type or reconstruction technique. Despite finding a significantly higher postoperative lymphocele rate in the multiple vessel cohort (*p* = 0.024), the authors concluded that MRA allografts can be safely used in pediatric renal transplantation with equivalent perioperative complication rates and graft survival outcomes to single artery allografts. De Coppi et al (5) reported 72 (29.9%) cases of vascular anomalies in a cohort of 241 pediatric cases, and no statistically significant differences were encountered when comparing MRA vs. with single renal artery (SRA) allografts with regard to vascular complications. However, a difference in mean creatinine values was encountered when comparing standard anatomy (control group with single artery, single vein) vs. groups with MRA (*p* < 0.01). Specifically, the SRA group had lower serum creatinine levels when compared to the groups with MRA at 1 year, but these differences disappeared by 5 years posttransplant. The authors attributed their results to an increased warm ischemic time (WIT) in the group with MRA, an important factor that highlights the importance of creating a single orifice when faced with MRA with efforts to minimize WIT by anastomosing a single lumen to the recipient artery (6).

The objective of this study was to perform a comparative retrospective analysis of pediatric kidney transplant recipients who received MRA vs. SRA allografts, with a surgical intent to create a single orifice (*via* vascular reconstruction when necessary) in as many MRA cases as possible, evaluating the occurrences of vascular, urologic, and surgical complications and overall clinical outcomes. Given that there have been relatively few reports of pediatric kidney transplants in which donor kidneys with MRA were utilized, we were interested in comparing our overall results with those reported by others.

## Materials and methods

This retrospective study aims to evaluate the clinical outcomes of pediatric kidney transplant recipients of MRA vs. SRA allografts. All pediatric recipients (aged 18 years or less) who underwent KT by a single surgeon at this center between January 2015 and June 2022 were included. Again, the surgical approach for both MRA and SRA allografts was to create a single arterial inflow using vascular reconstruction techniques when required, thereby minimizing WIT in the MRA cases, and without the routine use of surgical drains or ureteral stents. This study was approved by the Institutional Review Board at the University of Miami and follows the Helsinki Declaration (as revised in 2013). Written informed consent for transplantation was required and obtained from all subjects’ parents or a legal surrogate. Last follow-up date for the study was September 15, 2022.

### Pre- and post-transplant workup

All pediatric patients were evaluated by a pediatric transplant nephrologist and transplant surgeon prior to transplantation. They underwent a complete workup including laboratory studies (complete blood cell count, comprehensive metabolic panel, electrolytes, and serologies), radiographic imaging with echocardiogram, ultrasound of the abdomen, chest x-ray, and electrocardiogram (ECG). Currently, at our institution, we do not have cut-offs for recipient weight and height. Each patient is evaluated individually, and decisions were made in a multidisciplinary approach. All recipients began aspirin 81 mg daily on postoperative day 3 and remained on this regimen indefinitely. To monitor development of vascular and/or urological complications, baseline Doppler Ultrasound (DU) was performed immediately after surgery, and then repeated at 1, 3, and 12 months postoperatively. If there were any vascular or urological concerns, further imaging with magnetic resonance angiography and/or Tc99m MAG-3 renal scintigraphy was performed.

### Study groups

Patients were categorized into two groups according to presence of MRA or SRA of the donor kidney allograft. Group 1 was defined as the pediatric recipients that underwent a kidney transplant of an allograft with MRA—in each of these cases, back table reconstruction was required. Group 2 was defined as recipients that underwent a kidney transplant with an allograft having a SRA; none of these cases required any back table reconstruction prior to implantation.

### Surgical technique

A modified Gibson incision was made on the right lower quadrant and carried through the abdominal layers. The peritoneum was reflected medially, and the iliac vessels were exposed. Minimal possible dissection was performed, just enough to allow for vascular clamping of the vessels, avoiding unnecessary injury to the lymphatic system and subsequent lymphocele development. Based on the recipient's size, a liver and native right kidney mobilization was performed in an effort to accommodate an adult renal allograft (7). Depending on the recipient's size and length/diameter of vessels, vascular anastomosis was performed to the iliac vessels for larger recipients and for smaller children anastomosed to common iliac artery (CIA) or aorta and to the inferior vena cava (IVC) for venous drainage.

The renal allografts were prepared on the back table. Single or multiple renal arteries were identified and cleaned from surrounding tissue. The renal vein was isolated. If a right kidney from a deceased donor was used, the cava was reconstructed to increase the length of the right renal vein using 6-0 polypropolene sutures. Nontraumatic vascular clamps were placed. The allograft was brought to the field; we routinely perform the arterial anastomosis first using 7-0 polypropylene sutures, followed by the venous anastomosis with 6-0 prolene. Restoration of blood flow to the graft was performed followed by positioning of the kidney in the retroperitoneum, sitting on top of the right psoas muscle. Finally, the ureter was anastomosed to the bladder using a Miami Transplant Institute (MTI) ureteral technique (8), and the abdominal wall was closed in layers. It was not routine to use ureteral stents or Jackson–Pratt drains, unless very selectively indicated (8, 9).

### Vascular reconstruction of grafts with multiple renal arteries

**Figure 1:**
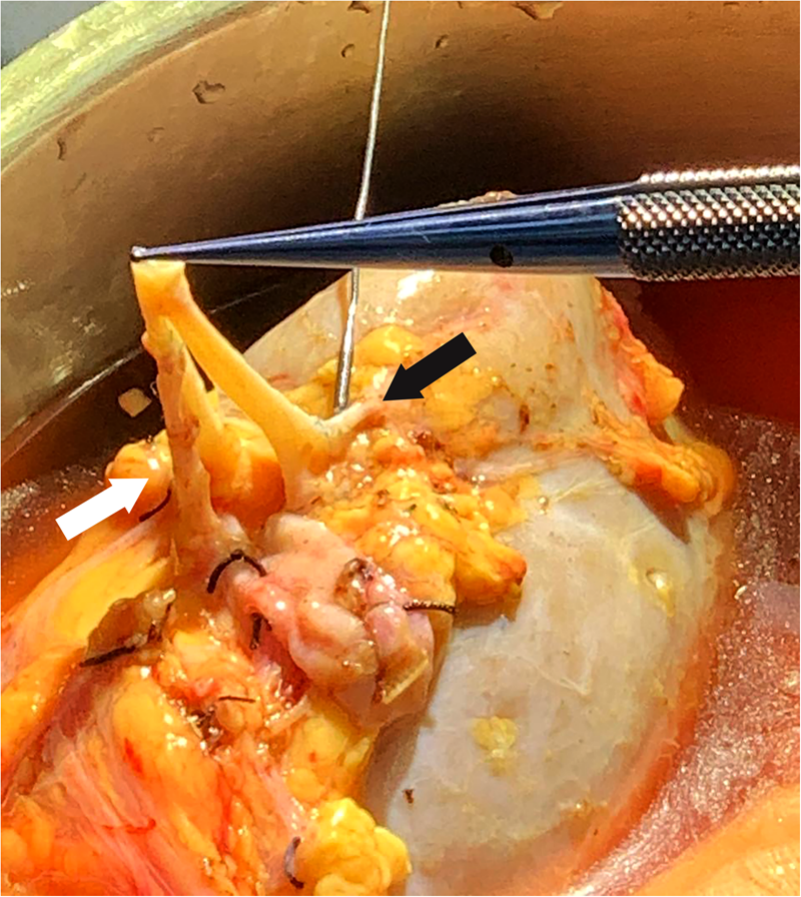
Adult allograft with two renal arteries reconstructed into a single orifice using polypropolene 8-0 and 2.5× loupes.

**Figure 2:**
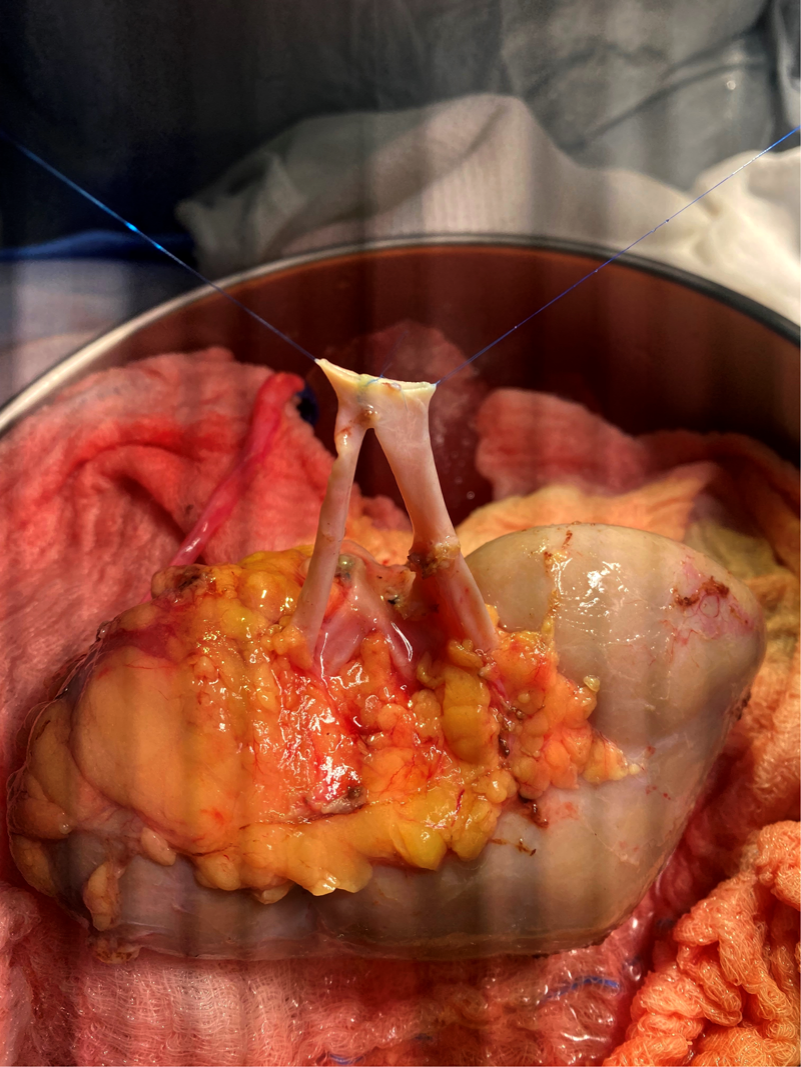
Adult allograft with three renal arteries, the lower pole RA was anastomosed end-to-side to the main RA (white arrow), and an upper pole RA end-to-side to a branch of the main RA inside the hilum (black arrow). RA, renal artery.

*Ex vivo* reconstructions were performed during bench surgery according to the case-specific anatomy utilizing surgical loupes at 2.5× magnification. All of the vascular reconstructions were performed with 8-0 prolene. Five of the grafts with MRA were from living donors and underwent reconstruction by creating a single orifice in order to decrease warm ischemia time (9). Reconstructions were done by either anastomosing two renal arteries of similar size side-to-side (Figure 1) or anastomosing accessory smaller renal arteries to the main renal artery in an end-to-side fashion (Figure 2). In one case, the ipsilateral inferior epigastric artery of the recipient was used as an interposition graft. Four grafts from deceased donor allografts had MRA that were kept with the aortic patch and anastomosed together to create a single large Carrel patch. Of these, one of the grafts was a horseshoe kidney that was divided at the isthmus on the back table under cold temperature; the left kidney was utilized, and it contained two RA with aortic patches that were conjoined.

### Baseline variables and clinical outcomes

Baseline variables that were studied included donor and recipient demographics, clinical characteristics of the underlying disease of recipients, donor kidney anatomic pathological evaluation, and pre- and intraoperative data, including history of surgical intervention pretransplant, history of bladder augmentation, type of arterial and venous anastomosis, WIT, cold ischemia time (CIT), and estimated blood loss (EBL). Serum creatinine was obtained at 6- and 12-months posttransplant. eGFR was calculated using Schwartz's original formula, where the constant of proportionality (k) is adjusted for the child's age and sex (10). Acute rejection was diagnosed after a clinically indicated renal allograft biopsy was evaluated by an experienced pathologist. All acute rejection episodes were treated based on type of rejection and constituted use of single agent or combination of them including pulse steroids, antithymocyte globulin, rituximab, total plasma exchange (TPE), and IV immunoglobulin. Delayed graft function (DGF) was defined as the need for dialysis during the first 7 days posttransplant. Graft primary nonfunction (PNF) was determined as ongoing DGF that required ongoing dialysis dependency after 3 months posttransplant. Complications (vascular or urologic) that developed within 30 days of kidney transplant were included and classified based on previously published Dindo–Clavien classification (11). Recipient outcomes including the development of postoperative complications, biopsy-proven acute rejection, renal function, and graft and patient survival were collected and analyzed.

### Statistical analysis

Baseline variables were described using frequency distributions for categorical variables, and means and standard errors for continuous variables (geometric means and corresponding standard errors were used for skewed distributions). Medians and ranges of continuous variables were also provided. Tests of association with the likelihood of having MRA (yes/no) vs. SRA were performed using Pearson chi-squared tests for categorical variables, standard two-sample *t*-tests for continuous variables, and log-rank tests for time-to-event variables. For skewed distributions, *t*-tests comparing geometric means were performed using natural logarithmic transformed values. *P*-values < 0.05 were considered to be statistically significant.

## Results

A total of 49 pediatric transplant recipients were included in the analysis. Of these, 9 had donors with MRA and were characterized as Group 1, and 40 recipients receiving a donor kidney with SRA were characterized as Group 2. We did not identify any significant differences when comparing both groups regarding the distributions of recipient age, sex, years on dialysis, height, weight, or diagnosis of end-stage renal disease (Table 1). Mean donor kidney length was slightly higher in Group 1 when compared to Group 2 (11.9 ± 0.4, *N* = 7 vs. 10.9 ± 0.2, *N* = 30; *p* = 0.04). There were no significant differences regarding distributions of living donor status, donor age, laterality, or percentage of glomerulosclerosis on biopsy, as demonstrated in Table 2.

**Table 1:** Associations of baseline recipient variables with donor multiple renal artery status.

Baseline variable	Percentage with characteristic for categorical variables; mean ± SE for (geometric mean */ SE for skewed) continuous variables		
	Donor MRA (*N* = 9)	Donor SRA (*N* = 40)	*p*-value
Recipient male	55.6% (5/9)	60.0% (24/40)	0.81
Recipient age (y)	13.0 ± 1.7	10.1 ± 0.9	0.16
Received RRT	88.9% (8/9)	75.0% (30/40)	0.37
Years on RRT (0 if none)	1.6 ± 0.5	1.9 ± 0.3	0.58
Recipient height (cm)	139.3 ± 9.1	127.5 ± 4.5	0.26
Recipient weight (kg)	40.2 ± 6.0	31.6 ± 2.8	0.20
ESRD diagnosis			0.41
Renal dysplasia	11.1% (1/9)	25.0% (10/40)	
Urogenital anomaly	0.0% (0/9)	17.5% (7/40)	
Combined renal dysplasia/urogenital anomaly	22.2% (2/9)	7.5% (3/40)	
FSGS	11.1% (1/9)	7.5% (3/40)	
Glomerulonephritis	11.1% (1/9)	5.0% (2/40)	
Cystinosis	11.1% (1/9)	2.5% (1/40)	
Other	11.1% (1/9)	25.0% (10/40)	
Unknown	22.2% (2/9)	10.0% (4/40)	
Recurrent ESRD	0.0% (0/9)	5.0% (2/40)	0.49

MRA, multiple renal arteries; SRA, single renal artery; RRT, renal replacement therapy; ESRD, end-stage renal disease; FSGS, focal segmental glomerulosclerosis.

**Table 2:** Associations of baseline donor variables with donor multiple renal artery status.

Baseline variable	Percentage with characteristic for categorical variables; mean ± SE for (geometric mean */ SE for skewed) continuous variables		
	Donor MRA (*N* = 9)	Donor SRA (*N* = 40)	*p*-value
Kidney length (cm)	11.9 ± 0.4 (*N* = 7)	10.9 ± 0.2 (*N* = 30)	0.04
Kidney volume (cm3)	152.3 ± 10.4 (*N* = 3)	155.3 ± 8.3 (*N* = 22)	0.90
Received LDKT	55.6% (5/9)	35.0% (14/40)	0.25
LD relation			0.72
Father	20.0% (1/5)	28.6% (4/14)	
Mother	40.0% (2/5)	50.0% (7/14)	
Other	40.0% (2/5)	21.4% (3/14)	
Donor age (y)	33.8 ± 4.3	28.3 ± 1.7	0.18
Right donor kidney graft	22.2% (2/9)	47.5% (19/40)	0.17
Kidney graft single vein	100.0% (9/9)	97.5% (39/40)	0.63
Kidney biopsy: % of GS	2.3 ± 1.2 (*N* = 6)	3.3 ± 1.2 (*N* = 31)	0.72

MRA, multiple renal arteries; SRA, single renal artery; LDKT, living donor kidney transplant; LD, living donor; GS, glomerulosclerosis.

Regarding perioperative and surgical technique variables, liver/native kidney mobilization was performed in 44.4% (4/9) of the cases in Group 1 and 60.0% (24/40) in Group 2 (*p* = 0.39) (Table 3). Of note, while the majority of these 28 recipients who received liver/native kidney mobilization were younger patients, three of them were 13–17 years of age at transplant. These three patients still required liver/native kidney mobilization because they had received donor kidneys that were larger in size compared with the donor kidneys received by the younger patients (results not shown). The target for arterial anastomosis was the CIA in 33.0% (3/9) of the cases for Group 1 and 60.0% (24/40) for Group 2. Regarding the venous anastomosis, the IVC was used in 44.4% (4/9) vs. 60.0% (24/40) for Groups 1 and 2, respectively. Neither of these differences were statistically significant. When comparing ischemia time, the mean warm ischemia time for the group with MRA was similar with 30.8 ± 1.9 vs. 30.3 ± 1.1 min for Group 2 (*p* = 0.85). The mean cold ischemia time was 10.0 ± 3.5 h for Group 1 and 15.6 ± 2.0 h for Group 2 (*p* = 0.22). Estimated blood loss was also not significantly different between the two groups (Table 3).

**Table 3:** Associations of perioperative and surgical technique variables with donor multiple renal artery status.

Baseline variable	Percentage with characteristic for categorical variables; mean ± SE for (geometric mean */ SE for skewed) continuous variables		
	Donor MRA (*N* = 9)	Donor SRA (*N* = 40)	*p*-value
L/NK mobilization	44.4% (4/9)	60.0% (24/40)	0.39
Surgical intervention pretransplant	44.4% (4/9)	62.5% (25/40)	0.32
Bladder augmentation	11.1% (1/9)	12.5% (5/40)	0.91
Arterial anastomosis with CIA	33.3% (3/9)	60.0% (24/40)	0.15
Venous anastomosis with IVC	44.4% (4/9)	60.0% (24/40)	0.39
WIT (min)	30.8 ± 1.9	30.3 ± 1.1	0.85
CIT (hr)	10.0 ± 3.5	15.6 ± 2.0	0.22
EBL (cc)	36.7 */ 1.4	22.1 */ 1.1	0.17

MRA, multiple renal arteries; SRA, single renal artery; L/NK, liver/native kidney; CIA, common iliac artery; IVC, inferior vena cava; WIT, warm ischemia time; CIT, cold ischemia time; EBL, estimated blood loss.

The clinical outcomes of both groups are described in Table 4. No cases of DGF or graft PNF were encountered. In addition, no vascular complications were observed in any of the patients. One urologic complication was observed for a single patient in Group 2 (none were observed in Group 1). This patient developed ureteral stricture at the ureterovesical junction with the development of acute kidney injury and readmission 2 weeks posttransplant requiring placement of a nephroureteral catheter by interventional radiology. The patient ultimately failed percutaneous intervention and required ureteral reimplantation approximately 7 months after his kidney transplant, classified as Clavien Grade 3b. Also of note, since there were no (vascular or urologic) complications observed in Group 1, there appeared to be no increased risk of developing a complication among the five MRA living donor kidney transplant (LDKT) recipients who required vascular reconstruction to achieve single orifice creation (vs. use of Carrel patch for the four deceased donor recipients with MRA).

**Table 4:** Associations of clinical outcomes with donor multiple renal artery status.

Outcome variable	Percentage with characteristic for categorical variables; mean ± SE for (geometric mean */ SE for skewed) continuous variables		
	Donor MRA (*N* = 9)	Donor SRA (*N* = 40)	*p*-value
Development of a postoperative complication (Clavien Grade ≥3)	0.0% (0/9)	2.5% (1/40)	0.63
Developed DGF	0.0% (0/9)	0.0% (0/40)	1.00
Experienced PNF	0.0% (0/9)	0.0% (0/40)	1.00
Developed an acute rejection	11.1% (1/9)	15.0% (6/40)	0.98^c^
Serum Cr at 6 months (mg/dl)^a^	0.7 */ 1.2 (*N* = 9)	0.7 */ 1.1 (*N* = 36)	0.68
Serum Cr at 12 months (mg/dl)^a^	0.9 */ 1.2 (*N* = 8)	0.7 */ 1.1 (*N* = 33)	0.46
eGFR at 12 months (ml/min/1.73 m^2^)^ab^	104.3 ± 11.6 (*N* = 8)	105.6 ± 4.7 (*N* = 33)	0.91
(Death-censored) graft failure	0.0% (0/9)	5.0% (2/40)	0.48^c^
Death with a functioning graft	0.0% (0/9)	2.5% (1/40)	0.71^c^
(Death-uncensored) graft loss	0.0% (0/9)	7.5% (3/40)	0.43^c^

MRA, multiple renal arteries; SRA, single renal artery; DGF, delayed graft function; PNF, primary nonfunction; Cr, creatinine.

^a^ Patients who developed graft failure (i.e., return to permanent dialysis) prior to the time point analyzed for serum Cr were not included in the calculation.

^b^ eGFR at 12 mo was calculated using Schwartz's original formula{=k * height at 12 mo/serum Cr at 12 mo, where k = 0.55 if age < 13 y or female, 0.70 if age > 13 y and male}.

^c^ Log-rank test p-value.

The geometric mean serum creatinine at 6 months posttransplant for Groups 1 and 2 were similar (0.7 */ 1.2, *N* = 9 vs. 0.7 */ 1.1, *N* = 36, *p* = 0.68), with similar results at 12 months posttransplant (0.9 */ 1.2, *N* = 8 vs. 0.7 */ 1.1, *N* = 33, *p* = 0.46). Mean eGFR at 12 months posttransplant between the two groups was also similar (*p* = 0.91) (Table 4). In addition, among the nine MRA recipients, mean serum creatinine at 6 and 12 months, along with mean eGFR at 12 months posttransplant, was not significantly different between the five LDKT and four deceased donor recipients (*p* = 0.81, 0.52, and 0.37, respectively; mean values not shown).

Among the 46 patients who were alive with a functioning graft at last follow-up, median follow-up was 24.2 (range: 2.4–72.7) months posttransplant. There were two cases of (death-censored) graft failure observed in the donor SRA group related to acute rejection (triggered by overt nonadherence in taking the prescribed immunosuppressive medications in one case). One case of death with a functioning graft was observed in the same group (Group 2) due to a cardiovascular event at home causing severe anoxic brain injury. The rates of (death-censored) graft failure and death with a functioning graft were not significantly different between the two groups (*p* = 0.48 and 0.71, respectively).

## Discussion

Kidneys with MRA are not an uncommon anatomic variation and when used as allografts have historically been associated with a higher incidence of vascular complications (12, 13). In our study, we demonstrated similar renal function at 6 and 12 months posttransplant without an increased rate of vascular or urologic complications in patients who received an allograft with MRA when compared to recipients of SRA allografts. In addition, while the sample sizes were small, there appeared to be no increased rate of complications among MRA recipients who required back table vascular reconstruction of their living donor grafts in order to achieve a single orifice.

Data comparing MRA vs. SRA outcomes in the pediatric population are scarce; thus, most of the pediatric outcomes are compared with data from the adult population. Kadotani et al (14). published a comparison between 292 transplants with SRA vs. 48 with MRA and identified higher incidence of vascular complications in the MRA group as well as a higher incidence of acute tubular necrosis (ATN) due to prolonged total ischemia time related to multiple vascular anastomosis, although this difference was not statistically significant (*p* = 0.45), likely due to the relatively low sample size in the MRA group. Similar results were reported in other publications (13, 15). O’Kelly et al. (3) published a retrospective cohort with 90 pediatric kidney transplants with MRA compared to 289 pediatric cases with SRA, and no significant differences regarding posttransplant graft loss, perioperative complications, or estimated GFR at 1 month or at 1 year posttransplant were encountered; however, a significantly higher incidence of lymphocele development was observed in the MRA cohort. While the cause of this finding was unclear, it was believed to be a result of higher recipient vessel skeletonization or more aggressive allograft hilar dissection (3). We did not encounter a similar complication rate which is likely due to utilizing different techniques during backbench preparation of the allograft. We routinely use electrosurgical bipolar vessel sealers when carefully dissecting the lymphoid tissue in the hilum that can potentially mitigate this risk.

We encountered a urologic complication for a single patient in Group 2, which was likely an ischemic insult to the ureter causing stricture at the ureteropelvic junction. Despite several nephroureteral stents and dilations, this was not successful, and a ureteral implantation in the operating room was performed at approximately 7 months posttransplant. This complication was classified as Clavien Grade 3b (11), and the recipient had no further issues. Our combined cohort urologic complication incidence was 2.0% (1/49), which falls close to that previously reported by Ciancio et al. (8) of 1.4% (7 events) among 500 consecutive kidney transplants without the use of ureteral stents. We do not believe that ureteral stents would have prevented this complication and, in fact, might add to patient risk of developing a urinary tract infection and need for performing further procedure(s) in the recipient.

A number of study limitations existed here. First, as this was a retrospective study with relatively small sample sizes (e.g., there were only nine recipients of MRA allografts), a randomized trial with a larger sample size would clearly be the gold standard for making more reliable statistical comparisons. In our study, good statistical power only existed for detecting large differences between Groups 1 and 2, as well as between LDKT recipients of MRA grafts who required vascular reconstruction to achieve a single orifice vs. deceased donor recipients of MRA grafts who did not require such vascular reconstruction. Despite this study limitation, we observed no vascular or urological complications among any of the MRA recipients. Second, this was an evaluation of pediatric transplant recipients performed at a single center by a single, highly experienced transplant surgeon. While one might expect that the results achieved by a single transplant surgeon using the same surgical approach, i.e., to perform careful vascular reconstructions among LDKT grafts with MRA with the goal of achieving a single orifice in each case (along with the avoidance of surgical drain and ureteral stent placement in all cases), would be more homogenous in comparison with those achieved by multiple surgeons using “more conventional” approaches, the observed favorable results reported here (by a single transplant surgeon) does need to be verified by others.

The present study aims to strengthen the pediatric kidney transplant data pool regarding the use of grafts with MRA. The continuing disparity between organ pool availability and an increasing waiting list of pediatric patients with end-stage renal disease requests greater use of extended criteria donors while also decreasing the discard rate of renal allografts (3). As demonstrated in this paper, MRA grafts should still be utilized as long as meticulous backbench preparation of the graft and complex vascular reconstruction are performed in order to limit the incidence of vascular or urologic complications from developing posttransplant. We did not encounter a significant increase in cold ischemia time or warm ischemia time due to the presence of MRA. Nieto-Ríos et al. (16) reviewed 347 renal transplants and found that CIT >12 h was an independent risk factor for developing DGF with a prevalence up to 18.4% in their study. We did not encounter any case of DGF here, which may be partially related to the use of hypothermic machine perfusion, as all our deceased renal grafts were placed into a LifePort® renal preservation machine (Organ Recovery Systems, Itasca, IL, United States) and subsequently stored in hypothermia (2–4 °C) using kidney preservation solution (KPS-1) (17).

In summary, our study demonstrates that pediatric renal transplants with MRA can be safely performed and without the routine use of surgical drains or ureteral stents, achieving similar outcomes as SRA grafts and with a low complication rate.

## Data Availability

The raw data supporting the conclusions of this article will be made available by the authors, without undue reservation.
